# Predicting emerging and re-emerging disease outbreaks through internet search trends: An analysis from India

**DOI:** 10.3934/publichealth.2019.1.1

**Published:** 2019-01-07

**Authors:** Sudip Bhattacharya

**Affiliations:** Himalayan Institute of Medical Sciences, C-5/12, HIHT campus, Dehradun

**Keywords:** outbreaks, infectious disease, surveillance

Early detection is the key for the prevention and control of any communicable diseases. In India the Integrated Disease Surveillance Programme (IDSP) was launched in November 2004 for surveillance purpose. In this portal, most of the Indian districts report data for 22 epidemic prone notifiable diseases.

This one stop portal can also respond to the outbreaks through skilled and well equipped Quick Response Teams (QRTs) [1]. Presently under IDSP, the information is collected on three specified reporting formats, namely “S” (suspected cases/syndromic), “P” (presumptive cases) and “L” (laboratory confirmed cases). “S” form is filled by the health workers to report data on suspected cases/syndromes. “P” form is filled up by Medical Officers to report data on probable/clinically suspected cases. “L” form is designed to collect data on lab confirmed cases.

The obtained data flows from bottom to top via S, P, and L forms. i.e., community to national level. An outbreak is detected by this surveillance mechanism takes about 7 to 10 days, which is dangerous for rapidly spreading diseases like swine flu, bird flu, nipah virus etc. Therefore, supplementary system to this IDSP will play a great role in collecting timely data on epidemic prone communicable diseases which may minimize the influence of unprecedented outbreaks. An innovative surveillance mechanism for predicting disease outbreaks embedded with internet search behaviour of the population has currently emerged as a promising technique [2]. Now a days, internet use via cell phone in India is rapidly increasing [3],[4]. Recently, a large proportion of medical or health-related information are flowing to the cell phones via internet [5]. Recent data indicates that primary sources of information flows through the Internet in most of the communities [6]–[9].

Presently, big multinational companies are using search data (after data mining) for monitoring purposes as well as for marketing purpose. Among all, e-news, social network data, and blog data are used as the main source [2]. This whole procedure is commonly known as “nowcasting” [10]. This “nowcasting” process can help us to estimate the magnitude of outbreaks by near real-time in early stages of an outbreak. Additionally, this process can be embedded with the existing healthcare setup. Hence, this approach is quite useful in resource-poor countries where their health systems are already overburdened. It is established from various studies that “Google Trends” may be a useful instrument for disease surveillance [11],[12]. This instrument can act as an add on measure to the existing IDSP system for timely disease surveillance. A study conducted by Verma M et al. in India with an objective to evaluate the temporal correlation between Google Trends and conventional IDSP data in Haryana and Chandigarh (U.T.) [13].

Google search was conducted for febrile illness like malaria, dengue fever, chikungunya, and enteric fever for the abovementioned area in 2016. Those extracted data were compared with the IDSP data (only P form). Google search trends and IDSP reporting showed temporal correlation. A lag of −2 to −3 weeks was observed between google search trends and IDSP data.

That study had some limitations. 1) The study used only the ‘P’ form data of febrile cases as it is difficult to differentiate the fever cases reported in “S” form. 2) Also, “L” form data also was excluded due to delay in lab reporting. 3) External validity was also emerged as an important concern. 4) Due to cultural diversity in India there are language differences. As Indians, we also do use social media/transfer of e-data in our vernacular language. In that study only, English language was used for data retrieval, which underestimates the actual value/burden and thus error was occurred in the calculation of correlation. 5) As Google Trends does not provide data at intra-province level, the established correlation may not help to identify the hotspot of an outbreak or epidemic. 6) Varied strength of positive correlation was observed with all febrile illnesses which may not have much significance. 7) Besides Google search, people may communicate/retrieve data via various search engines like, SIRI, Bing, E- Explorer or they may use social media like Facebook, Instagram or Twitter etc. 8) Lastly, seasonal variation could not be identified as the data was collected for short period of time (1 year only).

Despite of limitations, this google based surveillance system can reinforce the existing IDSP system. This kind of outbreak predicting intelligence system may be experimented at the ground level covering the larger communities in resources constraint areas like India. Mathematical modelling techniques can be incorporated in the future studies for accurate forecasting of epidemics and outbreaks for better adjustment of confounders. Not only “Google search”, other portal sites like “E-explorer”, “SIRI”, “Bing” and other search engines can be used for data mining and disease surveillance in the future.

In spite of the massive potential of this application, this technique should be used as an add-on to the existing surveillance systems. As we are now used to work manually (filling IDSP forms), training and retraining (for filling digital forms) of the peripheral health workers are the key for successful implementation of this intelligence system. However, the results of this study hold promise in Indian scenario for forecasting of emerging and re-emerging diseases outbreaks in near future.
